# Supplementary material for: Multi‐dataset identification of innovative feature genes and molecular mechanisms in keratoconus
Source: J Cell Mol Med. 2024 Sep 19;28(18):e70079. doi: 10.1111/jcmm.70079 (PMC11412914; doi:10.1111/jcmm.70079)
Supplement: Supplementary file 2 — Material S2. [file JCMM-28-e70079-s002.zip › 7.GSEA analysis/1.ARL11/KEGG_PRIMARY_BILE_ACID_BIOSYNTHESIS.html]

Details for gene set KEGG\_PRIMARY\_BILE\_ACID\_BIOSYNTHESIS[GSEA]

|  || Dataset | ARL11.group.cls#h\_versus\_l |
| Phenotype | group.cls#h\_versus\_l |
| Upregulated in class | h |
| GeneSet | KEGG\_PRIMARY\_BILE\_ACID\_BIOSYNTHESIS |
| Enrichment Score (ES) | 0.4412481 |
| Normalized Enrichment Score (NES) | 1.0229522 |
| Nominal p-value | 0.42307693 |
| FDR q-value | 1.0 |
| FWER p-Value | 0.999 |
Table: GSEA Results Summary

  

Fig 1: Enrichment plot: KEGG\_PRIMARY\_BILE\_ACID\_BIOSYNTHESIS      
 Profile of the Running ES Score & Positions of GeneSet Members on the Rank Ordered List

  

| SYMBOL | TITLE | RANK IN GENE LIST | RANK METRIC SCORE | RUNNING ES | CORE ENRICHMENT || 1 | BAAT | na | 614 | 0.452 | 0.1857 | Yes |
| 2 | CYP8B1 | na | 1368 | 0.351 | 0.3219 | Yes |
| 3 | CYP7A1 | na | 2190 | 0.283 | 0.4254 | Yes |
| 4 | AMACR | na | 4496 | 0.183 | 0.4412 | Yes |
| 5 | HSD3B7 | na | 8148 | 0.089 | 0.3763 | No |
| 6 | CYP39A1 | na | 10334 | 0.056 | 0.3384 | No |
| 7 | CYP46A1 | na | 11346 | 0.043 | 0.3287 | No |
| 8 | SLC27A5 | na | 12940 | 0.028 | 0.2952 | No |
| 9 | SCP2 | na | 17655 | -0.008 | 0.1630 | No |
| 10 | HSD17B4 | na | 19202 | -0.018 | 0.1267 | No |
| 11 | CYP27A1 | na | 19243 | -0.019 | 0.1339 | No |
| 12 | CH25H | na | 24990 | -0.076 | 0.0025 | No |
| 13 | CYP7B1 | na | 28122 | -0.134 | -0.0274 | No |
| 14 | AKR1C4 | na | 28839 | -0.153 | 0.0208 | No |
| 15 | ACOX2 | na | 28878 | -0.154 | 0.0889 | No |
| 16 | AKR1D1 | na | 29648 | -0.177 | 0.1463 | No |
Table: GSEA details [plain text format]

  

Fig 2: KEGG\_PRIMARY\_BILE\_ACID\_BIOSYNTHESIS      
 Blue-Pink O' Gram in the Space of the Analyzed GeneSet

  

Fig 3: KEGG\_PRIMARY\_BILE\_ACID\_BIOSYNTHESIS: Random ES distribution      
 Gene set null distribution of ES for **KEGG\_PRIMARY\_BILE\_ACID\_BIOSYNTHESIS**
